# Modular Nanotransporters for Nuclear-Targeted Delivery of Auger Electron Emitters

**DOI:** 10.3389/fphar.2018.00952

**Published:** 2018-08-27

**Authors:** Alexander S. Sobolev

**Affiliations:** ^1^Institute of Gene Biology, Russian Academy of Sciences, Moscow, Russia; ^2^Faculty of Biology, Lomonosov Moscow State University, Moscow, Russia

**Keywords:** modular nanotransporters, subcellular drug delivery, nuclear medicine, Auger electron emitters, cell nucleus, cancer

## Abstract

This review describes artificial modular nanotransporters (MNTs) delivering their cargos into target cells and then into the nuclei – the most vulnerable cell compartment for most anticancer agents and especially for radionuclides emitting short-range particles. The MNT strategy uses natural subcellular transport processes inherent in practically all cells including cancer cells. The MNTs use these processes just as a passenger who purchased tickets for a multiple-transfer trip making use of different kinds of public transport to reach the desired destination. The MNTs are fusion polypeptides consisting of several parts, replaceable modules, accomplishing binding to a specific receptor on the cell and subsequent internalization, endosomal escape and transport into the cell nucleus. Radionuclides emitting short-range particles, like Auger electron emitters, acquire cell specificity and significantly higher cytotoxicity both *in vitro* and *in vivo* when delivered by the MNTs into the nuclei of cancer cells. MNT modules are interchangeable, allowing replacement of receptor recognition modules, which permits their use for different types of cancer cells and, as a cocktail of several MNTs, for targeting several tumor-specific molecules for personalized medicine.

## Introduction

Subcellular delivery systems have attracted growing attention of researchers (Jhaveri and Torchilin, 2016; Ulasov et al., 2018). There are several groups of drugs that can be efficiently delivered using these systems. For example, agents that are efficient only within a particular subcellular compartment (e.g., DNA in the nucleus). Another group comprises drugs capable of exerting their effects in different cell compartments, but there is a particular cell compartment that is most affected by particular drugs. Therefore, there are cell compartments in which localization of a particular drug requires a minimum dose. Most anticancer agents have this characteristic. Examples are photosensitizers, which are used for photodynamic therapy of some diseases, especially oncological diseases, radionuclides emitting short-range particles like AEEs, and many others.

A wide variety of approaches have been developed to achieve this demanding goal, including different types of antibodies, small molecules, block copolymers, peptides, etc., with varying degrees of success.

Our group has developed an alternative approach, MNTs for delivery of different drugs requiring transportation into specified subcellular compartments, such as the cell nucleus, based on an engineered polypeptide platform (Slastnikova et al., 2012b,c, 2017a,b Sobolev et al., 2016). This review focuses on MNT delivery of AEEs; delivery of photosensitizers and other molecules by MNTs has been reviewed earlier (Sharman et al., 2004; Glover, 2012; Simoes et al., 2015; Slastnikova et al., 2015; Ulasov et al., 2018).

From our viewpoint, MNTs are the most suitable vehicles for cytotoxic agents whose destructive action spreads over very short distances from their places of location. In other words, these distances should be not longer than the size of the subcellular compartment at which the MNT is targeted. AEEs are one of the best example of such agents: their range is less than 1 μm, usually several tens of nanometers (Falzone et al., 2012; Knapp and Dash, 2016). AEEs, in particular ^125^I, attracted attention in the early 1970s (Adelstein, 1993) because of surprisingly high cytotoxic effect when incorporated into DNA. The observed high cytotoxicity was due to production of 24.9 (on average) Auger and Coster-Kronig electrons (hereafter called simply Auger electrons) per decay, which is accompanied by well-known emission of γ-rays. Emitted by many radionuclides used in nuclear medicine for many years, Auger electrons have linear energy transfer, 10–25 keV/μm, close to that of alpha-particles (Holzwarth, 2011), and deposit their energy, 10^4^–10^7^ Gy/decay, very close to the decaying nuclide, mainly within several nanometers (Kassis and Adelstein, 2005). In the case when the AEE decays in the close proximity to DNA, it is sufficient to produce multiple double-strand breaks of DNA (Balagurumoorthy et al., 2008; Piroozfar et al., 2018) resulting in cell death. Thus, the key challenge that must be addressed in order to exploit AEEs clinically is creation of vehicles that specifically deliver the AEEs into the nuclei of target cancer cells in tumors within the organism.

## Approaches to Nuclear-Targeted Delivery of Auger Electron Emitters

There were several attempts to develop methods of AEE delivery into the nuclei of cancer cells, in order to eradicate them, based on exploiting native biomolecules or their precursors which normally either are components of DNA or locate very close to it or interact with close DNA neighbors in living cell.

Many earlier studies were performed with 5-[^125^I]-iodo-2′-deoxyuridine, [^125^I]-IdU, a thymidine analog. [^125^I]-IdU demonstrated very high cytotoxic activity *in vitro*. *In vivo* experiments gave encouraging results, and pilot clinical trials were carried out (Macapinlac et al., 1996) but they demonstrated low incorporation of radioactivity in tumor cells after injection of [^125^I]-IdU and [^131^I]-IdU in hepatic artery of patients, which was explained by relatively low percentage, 15–50%, of tumor cells, which were in S phase. Another problem that should be taken into account in this approach is a competition of administered [^125^I]-IdU with natural DNA precursor, thymidine, whose level should be low enough for efficient [^125^I]-IdU incorporation (Chi et al., 2001).

Interestingly, the earlier assumption, that AEEs should be incorporated into DNA in order to reveal their cytotoxicity, was not then supported experimentally (summarized by Kassis, 2003). Moreover, it was shown that AEEs can exert a pronounced cytotoxicity when delivered into the nuclei by several molecules that do not incorporate into DNA but locate in the nucleus. DNA in relaxed chromatin is more susceptible to AEE-induced damage than in condensed state (Terry and Vallis, 2012). These molecules are ligands of sex steroid receptors, antibodies against nuclear proteins, and ligands to cell surface receptors which are able to reach the cell nucleus like EGF. These approaches have been recently reviewed in detail (Hillyar et al., 2014; Aghevlian et al., 2017; Bavelaar et al., unpublished^1^, in this issue), so the reader is addressed to these reviews for more detailed information. Here, we will consider approaches with EGF and another EGFR ligands as well as with somatostatin and its analogs, because they turned out to be efficient and reached phase I of clinical trials (Valkema et al., 2002; Vallis et al., 2014). They are based on (1) a well-known overexpression of EGFR (Yewale et al., 2013; Haddad et al., 2017; Xu et al., 2017) and somatostatin receptors (Anthony et al., 2002; Lobachevsky et al., 2012) characteristic of several cancer types, permitting targeting at these surface receptors, and (2) ability of EGFR (Han and Lo, 2012; Bazzani et al., 2018) and somatostatin receptors (Hornick et al., 2000; Wang et al., 2003) to translocate into the cell nucleus together with their ligands/cargos. Nevertheless, both approaches have some limitations. Concerning EGFR, normally only a relatively small part of internalized EGFR ligands reaches the cell nucleus. Approximately 7% of cell-bound EGF translocates into the cell nucleus during first 4 h of *in vitro* incubation of [^111^In]-DTPA-EGF with MDA-MB-468 human breast cancer cells; this index doubles by the 24th hour of incubation (Reilly et al., 2000). ^111^In-nimotuzumab, a humanized IgG_1_ anti-EGFR monoclonal antibody labeled with ^111^In, showed somewhat better nuclear accumulation in MDA-MB-468 cells: 16% of cell-bound ^111^In-nimotuzumab. Inclusion of a NLS into ^111^In-nimotuzumab almost doubled this index (Fasih et al., 2012), indicating that (1) natural nuclear translocation of EGF is not very efficient and (2) it is possible to find ways for improving nuclear delivery of AEEs by modifying vehicles based on receptor ligands. EGF administered *in vivo* demonstrated relatively low nuclear accumulation too: ca. 0.5% of administered EGF could be found in the nuclei of rat hepatocytes 1 h after intraportal administration of [^125^I]-EGF (Raper et al., 1987). We conclude that merely exploiting EGFR transport processes narrows this approach: (1) they can deliver only a relatively small portion of internalized EGFR cargo into the nuclei of target cancer cells, and (2) the spectrum of target cells is limited to only those that overexpress EGFR. There are no such limitations for MNTs: replaceable ligand modules permit targeting an MNT at almost any type of internalizable surface receptors, not only at EGFR (see section “Modular Nanotransporters: *in vitro* Delivery of Auger Electron Emitters” and “Modular Nanotransporters: *in vivo* Delivery of Auger Electron Emitters”). MNTs have an endosomolytic module that facilitates escape of the internalized MNTs from endosomes into the hyaloplasm and, thus, favor both interaction of the NLS-containing module of the MNT with importins and subsequent translocation of the MNTs into the nucleus (see section “Modular Nanotransporters: Principles and Structure”). This results in notably higher efficiency of nuclear translocation (Slastnikova et al., 2012b; Koumarianou et al., 2014). This in turn leads to significantly higher cytotoxic efficiency of AEE carried by MNTs than by EGFR ligands (see chapter “Modular Nanotransporters: *in vitro* Delivery of Auger Electron Emitters”). A similar situation can be observed with another peptide ligand to internalizable receptors, somatostatin and its synthetic analogs like octreotide. These are slowly internalizable molecules that can be partially translocated into the cell nuclei: not more than 10% of those internalized (Ginj et al., 2005; Eiblmaier et al., 2007). However, more recent publications indicate perinuclear localization of octreotide, and developed a special cleavable octreotide-cargo conjugates in order to facilitate nuclear delivery of the cleaved cargo (Lelle et al., 2015). Again, based on the similar grounds as for EGF/EGFR (see above), we believe that the MNT approach might achieve better results.

## Modular Nanotransporters: Principles and Structure

**FIGURE 1:**
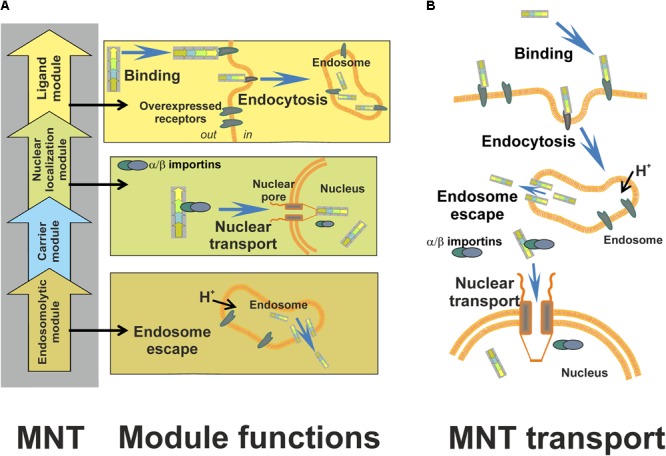
Schematic presentation of MNTs – their structure, functions, and subcellular transport [from (Slastnikova et al., 2017a) with permission]. **(A)** Principal scheme of MNTs, their structure and function of each module. **(B)** A scheme of MNT transport within the target cell. An MNT recognizes and binds to internalizable receptors, overexpressed on the target cancer cells, with the use of its ligand module; following subsequent receptor-mediated endocytosis, the endosomolytic module of the MNT performs escape from the endocytotic vesicles into the hyaloplasm; finally, the MNT is transported into the target cell nucleus due to its nuclear localization signal module. Ligand modules, MSH, EGF, and FA are targeted at melanocortin receptor-1, EGF receptor, and folate receptor, respectively. DTox is the endosomolytic module, the module with optimized SV-40 large T-antigen NLS is responsible for delivery into the nucleus of a target cell, and *Escherichia coli* HMP is a carrier module.

Modular nanotransporters designed for this purpose comprise: (1) an internalizable ligand module to provide MNT recognition of the target cell and subsequent receptor-mediated endocytosis of the MNT; (2) an endosomolytic module to enable the MNT to leave endosomes; (3) a module having the NLS to interact with importins, cytosolic proteins that ensure active transfer into the nucleus; (4) a carrier module (**Figure 1**). The terms “module” and “modular” are used here in their direct meanings, as MNT design considers the possibility of switching to various types of target cells and different subcellular compartments, which can be achieved more rapidly and easily if MNT components are readily exchangeable. The need for several (at least four) components is the following. First, MNTs can be endowed with cellular specificity simultaneously with the ability to penetrate inside the target cell if it comprises a component that has a high binding affinity to internalizable receptors. It is important that the receptors used be overexpressed on target cells and be weakly presented (in the ideal case, be absent) on normal cells. Second, specific nuclear delivery is achievable when the MNT has an NLS recognizable by importins. Third, importins are cytosolic proteins, whereas the MNTs, which enter the cell via receptor-mediated endocytosis, are enclosed in endocytotic vesicles (endosomes and others) and are thereby separated from the importins. This means that the MNTs enclosed in these vesicles cannot interact with the importins and need a special component to provide their escape from the endocytotic vesicles. Fourth, all components, or modules, should be integrated into a single transporter and should have an opportunity to attach the transported drugs to the transporter; the carrier module serves this purpose.

**FIGURE 2:**
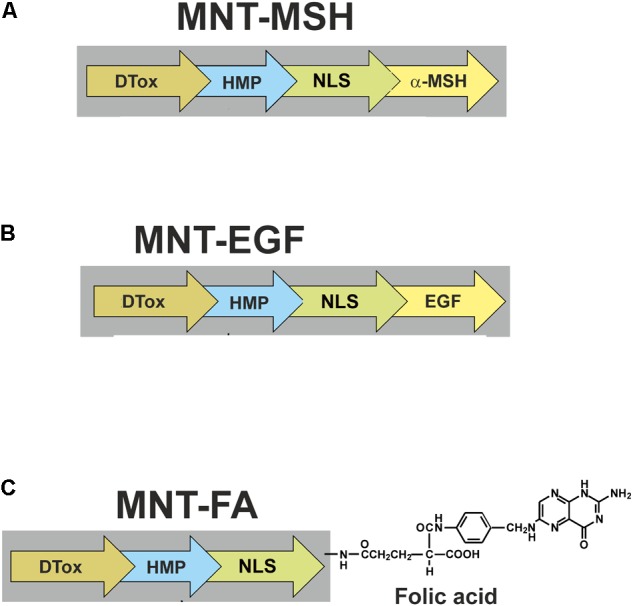
Scheme of MNTs discussed in this review. MNT targeted at **(A)** melanocortin receptors-1, **(B)** epidermal growth factor receptors, and **(C)** folate receptors. FA, folic acid.

For potential application, recombinant MNTs were bioengineered. Their structures and stepped penetration into a cell are schematized in **Figure 1**. They comprise: (1) either MSH (**Figure 2A**), or EGF (**Figure 2B**) as internalizable ligand modules that are targeted either at melanocortin receptors-1 overexpressed on melanoma cells (both human and murine), or at EGFRs overexpressed on bladder, esophageal, glioblastoma, head and neck, and several other types of cancer cells; (2) a modified NLS of the SV40 large T-antigen; (3) the DTox as the endosomolytic module; (4) HMP as a carrier (Rosenkranz et al., 2003a; Gilyazova et al., 2006). MNTs having other ligand modules were designed later: with interleukin-3 (this MNT targets acute myeloid leukemia cells with overexpression of interleukin-3 receptors), with somatostatin (for neuroblastoma cells with somatostatin receptor overexpression), and with folate (for cervical or ovarian cancer cells; **Figure 2C**) (Slastnikova et al., 2017a,b). MNTs are designated according to the sequence of their constituent modules from the N- to C-terminus, for example: DTox-HMP-NLS-MSH or DTox-HMP-NLS-EGF. The MNT molecule had molecular mass of ∼70 kDa depending on the MNT composition.

The modules in the MNT retain their functions within the chimeric MNT polypeptide. Equilibrium *K*_d_ for complexes of DTox-HMP-NLS-EGF with EGFRs was 29 nM (Gilyazova et al., 2006), a value close to the *K*_d_ for ^125^I-EGF. MSH, a short oligopeptide, had its affinity to melanocortin receptors slightly reduced when it was included into MSH-containing MNTs (to ∼20 nM) (Rosenkranz et al., 2003a).

The further fate of MNTs bound to internalizable receptors is predetermined by receptor-mediated endocytosis: the MNTs should appear in endosomes, which they should then actively leave to migrate to the hyaloplasm, where importins, which can bind to the NLS to provide MNT delivery to the nucleus, are located. The escape from endosomes should be fulfilled by the endosomolytic module, DTox, purposed to produce defects in membranes on the side where the pH is weakly acidic (as inside endosomes). We evaluated the ability of MNTs to generate pores in membranes by measuring dye leakage from dye-loaded liposomes. The tested MNTs caused dye leakage in two pH ranges (Rosenkranz et al., 2003b; Gilyazova et al., 2006; Khramtsov et al., 2008). One range, 5.5–6.5, is close to the endosomal pH and is due to the DTox module, as it can itself generate pores in this pH range. The other range was found at higher acidities, at pH values varying from 3 to 4, and it is ascribed to the effect of HMP.

The MNT-generated membrane defects found in experiments on liposomes were characterized electrochemically and by atomic force microscopy (Gilyazova et al., 2006; Khramtsov et al., 2008; Rosenkranz et al., 2008). The electrochemical study showed that after addition of the MNTs at pH 5.5, ion channels with conductivities of 2–5 nS appeared in planar lipid bilayers, whereas MNTs lacking an endosomolytic module did not show this effect. Channels did not appear when the full-sized MNT was added in a neutral medium (pH 7.0). In 5–15 min after the medium was acidified to pH of 5.5, atomic force microscopy revealed ring structures 30–50-nm in diameter in the lipid bilayer (egg lecithin) in the presence of the MNT. In 40–60 min, fluctuating holes having diameters of 50–200 nm and depths equal to the bilayer thickness were detected. At pH 7.0, such modifications were not detected. Biospecific atomic force microscopy with an anti-MNT antibody-modified cantilever tip showed that the elevations observed on the bilayer, which formed ring structures and were frequently seen near fluctuating pores, were built of MNT molecules (Khramtsov et al., 2008). These data provided additional characterization of the MNTs: analysis of the ring structures made it possible to estimate the average number of MNT molecules in those structures as 11 ± 2 (Slastnikova et al., 2012c). We concluded that upon acidification to 5.5, an MNT containing all the four modules can generate MNT-bordered pores in lipid bilayers whose sizes (50–200 nm) will be sufficient for the MNTs to escape through them.

The functionality of the endosomolytic module was confirmed at the cellular level by measuring intracellular pH in the microenvironment of the MNTs (Rosenkranz et al., 2003a). In experiments on living murine Cloudman S91 melanoma cells (clone M3), a truncated MNT version without the endosomolytic module, DTox, was detected in acidified vesicles, whereas the full-sized MNT was revealed in a neutral pH microenvironment.

Finally, to characterize the NLS-containing MNT module, interaction of the NLS with an α/β-importin dimer was assessed using surface plasmon resonance (Gilyazova et al., 2006): the affinity constants of the studied MNTs to the importin dimer were very close to the constant of the natural polypeptide with the same NLS, which implied that this module was fully functional.

The location of full-sized MNTs inside the cell was almost exclusively intranuclear (Gilyazova et al., 2006; Slastnikova et al., 2012c, 2017a,b). Thus, all modules in the MNT retained their intrinsic functions to make it possible to fulfill the major purpose, i.e., to deliver the MNTs into the nucleus of the target cell.

Further, the anticancer effects of AEEs carried by MNTs will be considered and discussed; their schemes are shown in **Figure 2**. One exception will be made for MNTs targeted to EGFR on bladder cancer cells: this will be the topic of a separate paper in this issue of this journal (Rosenkranz et al., unpublished^2^).

## Modular Nanotransporters: *In Vitro* Delivery of Auger Electron Emitters

As stated earlier, AEEs are most efficient in the cell nucleus, in the proximity of DNA, so the MNTs carrying AEEs into the nuclei of target cells are of special importance. Iodine-125, gallium-67, and indium-111, the isotopes emitting on average 24.9, 4.7, and 14.7 Auger electrons per decay, respectively (Howell, 1992), were used as MNT-delivered AEEs in our experiments. It was shown that ^111^In and ^67^Ga cause similar damage to plasmid DNA in solution (Othman et al., 2017).

The MNT containing EGF as its ligand module and carrying ^125^I (labeled by N-succinimidyl-4-guanidinomethyl- 3-[^125^I]iodobenzoate) accumulated in the nuclei of human epidermoid carcinoma A431 cells very efficiently: 60% of the radioactivity entering the cells appeared in the nuclei (Slastnikova et al., 2012b). The MNT-transported iodine-125 was 3500 times more cytotoxic to A431 human epidermoid carcinoma cells than [^125^I]-iodinated control polypeptide incapable of penetrating the cells (Slastnikova et al., 2012b). Cytotoxicity of [^125^I]-IdU expressed as *N*_37_, in decays per nucleus, can be used as a point of reference: i.e., as a maximum achievable cytotoxic efficiency of ^125^I because [^125^I]-IdU incorporates into DNA and its decays occur in it (Kassis et al., 1989). For Chinese hamster V79 lung fibroblasts, *N*_37_ is equal to 120 decays per cell nucleus (Kassis et al., 1989) and was obtained under the same experimental conditions utilized by Slastnikova et al. (2012b) for determining the cytotoxicity of [^125^I]-MNT on A431 cells. *N*_37_ in the latter report was estimated to be ca. 320 decays per nucleus: i.e., only 2.7 times less effective than ^125^I directly incorporated into DNA. Cytotoxicity of [^125^I]-MNT for D247.MG glioma cells measured as radioactivity per ml required to reduce cell survival to 37%, *A*_37_, was 9 μCi/ml (Slastnikova et al., 2012b), whereas that of [^125^I]-IdU, evaluated on the same cell line, was 3.1 μCi/ml (Larsen et al., 1997), giving almost the same ratio, 2.9, of relative efficiency as above. Similar results (Koumarianou et al., 2014) were obtained for gallium-67 transported with DTox-HMP-NLS-EGF (**Table 1**). In both these studies, the efficacy of MNT-delivered AEEs targeted at cancer cells with EGFR overexpression was compared with the efficacy of the same AEEs (^125^I and ^67^Ga) attached to EGF. The MNT-delivered ^125^I and ^67^Ga had cytotoxicities significantly higher than the EGF-delivered ^125^I and ^67^Ga (**Table 1**). We concluded that the mere binding to an EGFR and subsequent internalization are insufficient for providing high cytotoxicity of AEEs. Therefore, it is required to provide the AEE delivery vehicle with the possibility of escaping from endocytotic compartments and with the NLS, i.e., with the modules that are present in the MNT.

**Table 1:** Cytotoxicity of AEE-DTox-HMP-NLS-EGF compared with other AEE-delivering vehicles [after (Koumarianou et al., 2014; Slastnikova et al., 2012b)].

Cell line	Vehicle	AEE	*A*_37,vehicle_/*A*_37,MNT_^∗^	*A*_10,vehicle_/*A*_10,MNT_^∗^
A431 human epidermoid carcinoma	EGF	^67^Ga	13	18
	Bovine serum albumin	^67^Ga	17	17
	EDTA	^67^Ga	385	388
	Bovine serum albumin	^125^I	3,500	n.d.
	EGF	^125^I	5	18
U87MG.wtEGFR human glioblastoma	EGF	^67^Ga	72	18
D247MG human glioblastoma	Bovine serum albumin	^125^I	60	n.d.

^∗^Ratio of *A*_*37*_ and *A*_*10*_ values of vehicles to values for corresponding AEE-DTox-HMP-NLS-EGF (either with ^*67*^Ga or with ^125^I); *A*_*37*_ and *A*_*10*,_ radioactivity per well required to reduce cell survival to 37% or 10%, respectively. n.d., not determined.

The cytotoxicity of ^111^In-carrying MNTs compared to control ^111^InCl_3_ was enhanced significantly, about 160-fold (if one compares *A*_37_ doses). It was also shown that the cytotoxic potency of these ^111^In-MNTs was dependent on their specific activity. These effects were observed for ^111^In delivered by two types of MNTs: one, DTox-HMP-NLS-MSH, targeted at cancer cells overexpressing melanocortin receptors-1 (experiments on B16-F1 mouse melanoma cells) and another, DTox-HMP-NLS-EGF, targeted at EGFRs (EGFR; experiments on U87.wtEGFR human glioblastoma and A431 human epidermoid carcinoma cells) (Slastnikova et al., 2017a).

In normal tissues and non-activated macrophages, FRs are usually not exposed to agents circulating in the blood, whereas malignant transformation of cells or activation of macrophages make the FRs on these cells both overexpressed and accessible to the molecules dissolved in blood. These features of FRs made them a widely exploited target (Lu and Low, 2012; Xie et al., 2016; Srinivasarao and Low, 2017). The list of diseases with abnormal cells characterized by overexpression and/or accessibility of FRs includes several types of cancer and a group of diseases that are characterized by inflammation, e.g., rheumatoid arthritis, atherosclerosis, and Crohn’s disease (Slastnikova et al., 2015, 2017b).

An FR-specific MNT was created according to the following two-step procedure (Slastnikova et al., 2017b). First, a ligand-free MNT, DTox-HMP-NLS-Cys, with an additional cysteine residue on the MNT C-terminus was prepared. Second, the final folate-bearing MNT, hereafter called folate-MNT, was obtained by conjugation of folate-polyethylene glycol (3.4 kDa)–maleimide to the ligand-free MNT. Thus, produced folate-MNT (**Figure 2C**) demonstrated all the necessary properties including specific interaction with FRs, FR-dependent internalization, and accumulation within the nuclei of target cancer FR^+^ cells (human cervical carcinoma HeLa) in contrast to FR^-^ cells (human lung adenocarcinoma A549). Moreover, when an excess of free folate was added to FR^+^ cells, the cytotoxic efficiency of ^111^In carried by the folate-MNT sharply declined (Slastnikova et al., 2017a,b). These data clearly demonstrate that effects of the AEE-carrying MNTs are receptor-dependent and receptor-specific. The cytotoxic effect of ^111^In delivered by the folate-MNT was enhanced considerably in comparison with control ^111^In as was shown on FR-positive HeLa and U87MG.wtEGFR cells (Slastnikova et al., 2017b).

These data clearly demonstrated that MNTs can efficiently deliver AEEs into the nuclei of different target cells, where the AEEs reveal their cytotoxic effects. This conclusion is based on results obtained on five different cancer cell lines (B16F1 murine melanoma, A431 human epidermoid carcinoma, HeLa human cervical carcinoma, U87MG.wtEGFR, and D247MG human glioblastoma) with three different AEEs (^125^I, ^67^Ga, and ^111^In).

## Modular Nanotransporters: *In Vivo* Delivery of Auger Electron Emitters

Modular nanotransporter showed low acute and chronic toxicity in mice and rats, low immunogenicity/allergenicity in mice and guinea pigs, and they are non-pyrogenic to rabbits (Slastnikova et al., 2012c; Yakubovskaya et al., unpublished).

The selectivity of accumulation of labeled MNTs in B16-F1 tumor-bearing C57Black/6J mice reached the ratios 13.4 (tumor/muscle) and 9.8 (tumor/skin) 3 h after intravenous administration (Slastnikova et al., 2012a,c).

After being injected intravenously, DTox-HMP-NLS-MSH accumulated mainly within cancer cells of experimental tumors (Cloudman melanoma S91, clone M3) of DBA/2 mice. Its subcellular localization was predominantly nuclear. A similar result was obtained with another MNT, DTox-HMP-NLS-EGF, intravenously administered to Balb/c ByJIco-*nu/nu* mice bearing A431 human epidermoid carcinoma xenografts (Slastnikova et al., 2012c).

Folate-MNT-^111^In demonstrated significant intratumoral retention of ^111^In radioactivity after intratumoral injection: the decay-corrected retention half-life was 52 h for HeLa xenograft in contrast to control ^111^In-EDTA which was rapidly eliminated from the tumor with decay-corrected retention half-life of 24 min. Following intratumoral administration of folate-MNT-^111^In, normal tissues showed low radioactivity, which was mostly limited to liver and kidney (Slastnikova et al., 2017b). Intratumoral (B16F1 melanoma, C57BL/6J mice) retention of intratumorally injected ^111^In-DTox-HMP-NLS-MSH was even better: 3.7 days as fitted to a single exponential. Normal tissues again demonstrated nearly undetectable radioactivity that was observed only in liver and kidney (Slastnikova et al., 2017a).

Radioactivity dose-dependent (2.6, 5.2, and 10.4 MBq per mouse) tumor growth delay was observed in experiments with C57BL/6J mice bearing B16F1 melanoma tumors after a single intratumoral administration of ^111^In–DTox-HMP-NLS-MSH. 82% tumor growth inhibition, compared to control animals receiving saline, was observed at 10.4 MBq, which was the most efficient dose. Control ^111^In-EDTA or non-labeled ^111^In–DTox-HMP-NLS-MSH did not result in any tumor growth inhibition at the corresponding doses (Slastnikova et al., 2017a).

Intratumoral administration of folate-MNT-^111^In (7.5 and 15 MBq per mouse) also resulted in dose-dependent tumor growth delay. Fifteen megabecquerel was more efficient, demonstrating ∼80% tumor growth inhibition if compared with control animals (the same dose of ^111^In-EDTA or non-labeled MNT). The experimental group of mice (15 MBq of ^111^In–DTox-HMP-NLS-MSH intratumorally) showed 60% survival at day 93, whereas animals in control groups demonstrated 0% survival by the 21st and 30th day (Rosenkranz et al., 2017; Slastnikova et al., 2017b).

Auger electron emitters are considered as a promising cytotoxic agents for treating small-sized tumors (Shinohara et al., 2018) including those under hypoxic conditions (Weeks et al., 2010) like hypoxic regions are frequently found in glioblastoma (Yoshii et al., 2018). It is also important for attention to be given to the recently published theoretical paper of Raghavan et al. (2017). They suggest that AEEs carried by MNTs might be optimal for the treatment of infiltrative brain tumors like glioblastoma multiforme when delivered by convection enhanced delivery into margins of a surgically created resection cavity. They developed a model that identified an infusion protocol and optimal AEEs. The calculations suggest that a protocol containing convection-enhanced delivery of AEEs carried by MNTs is promising in order to achieve sufficient dose to destroy tumor cells within the 2-cm cavity margin (∼1 tumor cell per 10 normal cells) (Burger and Kleihues, 1989) with minimal dose to healthy cells at clinically practical radioactivity levels.

## Conclusion

Auger electron emitters carried by MNTs were shown to demonstrate high cytotoxicity for cancer cells and exhibit promising therapeutic potential in murine cancer models. These results suggest that MNTs deserve further evaluation as a platform technology for AEE radiotherapy. We also anticipate that the application of MNTs as delivery vehicles will increase the range of therapeutic radionuclides by including well-known diagnostic radionuclides (like ^111^In, ^67^Ga, etc.), for which methods for production and separation have been developed, and, thus, they will be able to be used in this new quality. A strong point of MNTs is the substitutability of their modules, offering a stimulating potential of generating an MNT cocktail with an optimal combination of ligand modules and subcellular localizing sequences tailored to the molecular profile of an individual patient’s tumor (Airley and Evans, 2012).

## Author Contributions

The author confirms being the sole contributor of this work and approved it for publication.

## Conflict of Interest Statement

The author declares that the research was conducted in the absence of any commercial or financial relationships that could be construed as a potential conflict of interest.

## Footnotes

1 Bavelaar, B. M., Lee, B. Q., Gill, M. R., Falzone, N., and Vallis, K. A. (2018). Subcellular targeting of theranostic radionuclides. *Front. Pharmacol.*

2 Rosenkranz, A. A., Slastnikova, T. A., Karmakova, T. A., Vorontsova, M. S., Morozova, N. B., Petriev, V. M., et al. (2018). Antitumor activity of auger electron emitter 111In delivered by modular nanotransporter for treatment of bladder cancer with EGFR overexpression. *Front. Pharmacol.*

## Abbreviations

*A*_37_ and *A*_10_: radioactivity per well required to reduce cell survival to 37% or 10%, respectively
AEEs: Auger electron emitters
DTox: translocation domain of diphtheria toxin
EDTA: ethylene diamine tetraacetic acid
EGF: epidermal growth factor
EGFR: EGF receptor
FRs: folate receptors
HMP: *Escherichia coli* hemoglobin-like protein
*K*_d_: dissociation constant
MNTs: modular nanotransporters
MSH: α-melanocyte stimulating hormone
*N*_37_: number of decays per nucleus required to reduce cell survival to 37%
NLS: nuclear localization sequence
